# Editorial: Pediatric acute lymphoblastic leukemia: what's next?

**DOI:** 10.3389/fped.2023.1358139

**Published:** 2024-01-10

**Authors:** J. V. Gil, J. Ribera, M. Llop

**Affiliations:** ^1^ Grupo de Investigación en Hematología y Hemoterapia, Instituto de Investigación Sanitaria la Fe, Valencia, Spain; ^2^ Acute Lymphoblastic Leukaemia Group, Josep Carreras Leukaemia Research Institute, Badalona, Spain; ^3^ Unidad de Biología Molecular, Hospital Universitari I Politècnic la Fe, Valencia, Spain

**Keywords:** acute lymphoblastic leukemia, overall survival, molecular characterization, surface markers, precision medicine

**Editorial on the Research Topic** Pediatric acute lymphoblastic leukemia: what’s next?

## Word limit consideration

Adhering to the prescribed word limit, this editorial concisely synthesizes the key themes and findings from the four contributing articles. The objective is to provide a comprehensive overview without being an exhaustive table of contents, respecting the guidelines that govern the editorial's length.

## Introduction

Acute lymphoblastic leukemia (ALL) is the most common hematological cancer in children. The survival rates have improved significantly in recent decades, and the current therapy strategies focus on limiting toxicities and improving outcomes in very high risk and relapsed patients. The benefit of emerging treatments such as targeted drugs and immunotherapy (antibodies, CAR-T-cells) is being explored, and thus basic and clinical research will provide the knowledge to better understand and manage pediatric ALL. This research topic aimed to discover what's next in ALL. This hematologic malignancy has been analyzed from different points of view.

## Contributing articles

A mini-review conducted by Stolpa et al. examined the prognostic role of CD34+CD38− lymphoblasts in B-cell precursor acute lymphoblastic leukemia (BCP-ALL) at the time of diagnosis. Despite the lack of statistically significant differences in the initial treatment response, the presence of CD34+CD38− lymphoblasts is identified as a potential unfavorable prognostic factor for disease recurrence. Similar studies have investigated the prognostic impact of specific cell surface markers in leukemia (1, 2). Beyond the assessment of specific cell populations at diagnosis, research on minimal residual disease (MRD) has consistently shown its significance in predicting treatment response and long-term outcomes in ALL (3).

An original research conducted by Terra Correa et al. focused on the overall and event-free survival rates of pediatric ALL patients in a Brazilian center. The study revealed that despite advancements in treatment, the survival rates are comparable with previous studies conducted in Brazil, which are lower than those observed in developed countries. The challenges in risk stratification, prognostic testing, and the impact of treatment-related adverse effects highlight the complexities in improving outcomes for pediatric ALL. Comparative studies from different regions have reported variations in survival rates and treatment outcomes. For example, a global analysis conducted by Hunger et al. (4) emphasized the disparities in survival rates for pediatric ALL on a global scale, pointing to the need for region-specific strategies and improvements in treatment protocols to enhance survival outcomes.

Velasco et al. provided a perspective introducing the Relapsed ALL Network (ReALLNet), a pioneering initiative that connects patient care with expert groups in relapsed or refractory ALL. This network, established under the European strategic plan, aims to facilitate interdisciplinary collaboration, preclinical research, and data collection to address the unique challenges posed by this patient population. Similar initiatives, such as the Children's Oncology Group (COG) in the United States and the UKALLR3 trial in the United Kingdom, have demonstrated the efficacy of collaborative networks in conducting clinical trials, sharing data, and advancing the understanding of relapsed ALL. In the same line, molecular platforms, such as the LEAP consortium project (5) in the United States and INFORM (6) in Germany, demonstrated that between 8% and 12% of patients with a relapsed malignancy can benefit from a targeted treatment based on oncogenomic studies guided by a multidisciplinary national board. These networks play a crucial role in providing precision medicine in pediatric oncology.

Onyije et al. presented a viewpoint regarding the risk factors associated with childhood ALL, which are important for identifying modifiable factors for effective prevention. Noteworthy findings include the association between maternal exposure to pesticides and childhood ALL risk, as well as the upgraded evidence on childhood exposure to domestic radon. These updates contribute to our ongoing efforts to understand and mitigate the risk factors associated with childhood ALL. Similar findings have been reported in meta-analyses and systematic reviews. For instance, a meta-analysis conducted by Bailey et al. (7) supported the association between maternal pesticide exposure and childhood leukemia risk. In addition, studies on environmental factors, including radon exposure, have indicated their potential role in developing leukemia, contributing to the ongoing efforts to identify modifiable risk factors (8, 9).

In conclusion, the research topic “Acute Lymphoblastic Leukemia: What's Next” exemplifies the dynamic nature of ALL research, presenting findings that propel us toward a deeper understanding of the disease and its management.

## References

[1] Jiang Z Wu D Lin S Li P. CD34 and CD38 are prognostic biomarkers for acute B lymphoblastic leukemia. Biomark Res. (2016) 4(1):1. 10.1186/s40364-016-0080-5 28018598 PMC5159997

[2] Blatt K Menzl I Eisenwort G Cerny-Reiterer S Herrmann H Herndlhofer S Phenotyping and target expression profiling of CD34+/CD38− and CD34+/CD38+ stem- and progenitor cells in acute lymphoblastic leukemia. Neoplasia. (2018) 20(6):632–42. 10.1016/j.neo.2018.04.004 29772458 PMC5994777

[3] Pui CH Yang JJ Hunger SP Pieters R Schrappe M Biondi A Childhood acute lymphoblastic leukemia: progress through collaboration. J Clin Oncol. (2015) 33(27):2938–48. 10.1200/jco.2014.59.1636 26304874 PMC4567699

[4] Hunger SP Lu X Devidas M Camitta BM Gaynon PS Winick NJ Improved survival for children and adolescents with acute lymphoblastic leukemia between 1990 and 2005: a report from the children’s oncology group. J Clin Oncol. (2012) 30(14):1663–9. 10.1200/jco.2011.37.8018 22412151 PMC3383113

[5] Walter RB Michaelis LC Othus M Uy GL Radich JP Little RF Intergroup LEAP trial (S1612): a randomized phase 2/3 platform trial to test novel therapeutics in medically less fit older adults with acute myeloid leukemia. Am J Hematol. (2017) 93(2):49–52. 10.1002/ajh.24980 PMC5760282 29164656

[6] Van Tilburg CM Pfaff E Pajtler KW Langenberg KP Fiesel P Jones BC The pediatric precision oncology INFORM registry: clinical outcome and benefit for patients with very high-evidence targets. Cancer Discov. (2021) 11(11):2764–79. 10.1158/2159-8290.cd-21-0094 34373263 PMC9414287

[7] Bailey HD Infante-Rivard C Metayer C Clavel J Lightfoot T Kaatsch P Home pesticide exposures and risk of childhood leukemia: findings from the childhood leukemia international consortium. Int J Cancer. (2015) 137(11):2644–63. 10.1002/ijc.29631 26061779 PMC4572913

[8] Kendall GM Wakeford R Bunch K Vincent TJ Little MP. Residential mobility and associated factors in relation to the assessment of exposure to naturally occurring radiation in studies of childhood cancer. J Radiol Prot. (2015) 35(4):835–68. 10.1088/0952-4746/35/4/835 26512630

[9] Kendall GM Little MP Wakeford R. A review of studies of childhood cancer and natural background radiation. Int J Radiat Biol. (2021) 97(6):769–81. 10.1080/09553002.2020.1867926 33395329 PMC10686050

